# Proportion of people identified as transgender and non-binary gender in Brazil

**DOI:** 10.1038/s41598-021-81411-4

**Published:** 2021-01-26

**Authors:** Giancarlo Spizzirri, Raí Eufrásio, Maria Cristina Pereira Lima, Hélio Rubens de Carvalho Nunes, Baudewijntje P. C. Kreukels, Thomas D. Steensma, Carmita Helena Najjar Abdo

**Affiliations:** 1 Department of Psychiatry, Faculdade de Medicina FMUSP, Universidade de Sao Paulo, São Paulo, SP Brazil; 2 Independent Researcher, São Paulo, SP Brazil; 3 Department of Neurology, Psychology and Psychiatry, Botucatu Medical School, Universidade Estadual Paulista (UNESP), Botucatu, SP Brazil; 4 Programa de Pós-Graduação em Enfermagem, Universidade Estadual Paulista (UNESP), Botucatu, SP Brazil; 5 Department of Medical Psychology, Center of Expertise On Gender Dysphoria, Amsterdam University Medical Centers, Location VU, Amsterdam, The Netherlands

**Keywords:** Health policy, Epidemiology

## Abstract

Studies estimate that gender-diverse persons represent 0.1 to 2% of populations investigated, but no such assessment was performed in Latin America. In a representative sample of Brazil’s adult population (n = 6000), we investigated participants' sociodemographic characteristics and possible associations between these and current gender identity, categorized as cisgender, transgender or non-binary gender. We also investigated transgender individuals' distress associated with gender-related body characteristics. As main results, we found that transgender individuals represented 0.69% (CI95% = 0.48–0.90) of the sample, whereas non-binary persons were 1.19% (CI95% = 0.92–1.47). These percentages were not different among Brazil’s 5 geographic regions. Preliminary analyses showed that transgender individuals were on average younger (32.8 ± 14.2 years, CI95% = 28.5–37.1), compared to cisgender (42.2 ± 15.9, CI95% = 42.5–42.8) and non-binary (42.1 ± 16.5 years, CI95% = 38.3–46.5) groups. Non-binary persons are less likely to be in a relationship compared to cisgender individuals (OR = 0.57, CI95% = 0.35–0.93). In the transgender group, 85% of transgender men and 50% of transgender women reported distress due to gender-related body characteristics. Our main findings draw attention that gender-diverse Brazilian individuals represent around 2% of the country's adult population (almost 3 million people), and are homogeneously located throughout the country, reiterating the urgency of public health policies for these individuals in the five Brazilian sub-regions.

## Introduction

Transgender (TG) is an umbrella term that describes people who identify with a gender that is incongruent or different from the one assigned to them at birth^1^. Non-binary gender (NBG), on the other hand, is an umbrella term that describes those who feel their gender identity is outside or in between male and female identities, e.g. a person who experiences both identities or neither^2^. The boundaries among the categories of gender identity often overlap. For instance, the term gender-nonconforming (GNC) describes individuals whose gender identity, role, or expression differs from what is expected for the socially accepted genders (usually, man or woman) in a given culture and given time^3^. Gender assigned at birth is generally based on physical sex characteristics in a binary way: male individuals are assigned men, whereas female individuals are assigned women^1^.

The estimated proportion of gender-diverse individuals (those who are not cisgender) varies between 0.1 and 2% of the population, depending on the inclusion criteria and where the studies were held^4^. Most epidemiological studies on gender-diverse people assessed individuals who were already treated or referred for treatment at gender-affirming healthcare centers^5^. However, available data may underestimate the number of people with gender incongruence as many are reluctant to seek help. This may be due to shame, self-esteem issues, current social morals influencing hostile treatment against them, financial problems, lack of support or for not being aware of their own gender-diverse identity. The risk of losing family support, work and relationships also have an impact on the decision to seek specialized help^5–8^, which may be especially hard for those living in areas where discrimination is the norm^9^. It is also worth noting that the proportion of individuals identifying as gender-diverse exceeds the estimated number of people who receive gender-affirming medical assistance^3,5,10^, because not all gender-diverse individuals desire medical interventions.

Only a few studies identify the proportion of gender diversity in the general population based on self-report^11^. Apart from estimating the proportion of gender-diverse people, a study shows an increase in the number of US citizens who identify as TG in the last decade^12^. They also indicate the importance of both informing the population about gender diversity^3^ and performing research that is conducted in samples that are diverse regarding age, race/ethnicity, and socioeconomic status^13^. There is evidence that low educational and socioeconomic levels have negative health implications on the TG population^14^. Studies also highlight the necessity for school counselors and health professionals to be prepared to provide the support needed for this group of individuals^15^.

TG people in many countries live in the margins of society^9^. They suffer discrimination, social stigma, exclusion and often have a traumatic life. They have little access to education and work, usually belong to lower social classes, have less access to healthcare and have shorter lifespans^9^. A US study showed that TG individuals have lower socioeconomic status despite of having higher education levels^16^. However, a recent study found that the group’s education attainment is lower than their cisgender counterparts^17^. Between 2008 and 2018, Latin America had the highest incidences of violence against TG people in the world. Seventy-eight percent of all murders targeting this population occurred in Latin America^18^, and most of them in Brazil^19^.

It is essential to measure the proportion of gender-diverse people in the population, in order to drive and inform public health policies and raise awareness on matters that affect these individuals. This is the first population-based study representing Brazilian adults, aiming to estimate the number of gender-diverse individuals in the country; as well as to preliminarily describe their sociodemographic characteristics and self-reported distress related to their bodies.

## Methods

### Study design and population

Brazil is a large country, with 26 states (each with its own capital city and metropolitan area), and a federal district. The states are grouped in five geographical regions (North, Northeast, Midwest, Southeast and South). The population is distributed as follows: North 8%, Northeast 26%, Midwest 7%, Southeast 44%, and South 15%. Fifty-eight percent of Brazilians live in metropolitan areas, whereas 42% live in the countryside. The estimated adult population in December 2018 was 158,000,000 people^20^.

The present cross-sectional study was part of a larger assessment conducted by DataFolha Research Institute in Brazil between November and December 2018.

The questionnaire was divided into two parts: the first considered sociodemographic characteristics (General Instrument, GI), whereas the second assessed specific aspects of one’s gender identity (Specific Instrument, SI), as described in detail in the section “Measures”.

Before starting the main data collection, a pilot test with 45 individuals (8 of which were transgender) was performed to assess how long it took to answer both instruments (GI and SI), as well as potential inconsistencies in the SI. The time to finish SI was approximately 4 min and answering both instruments did not exceed 15 min.

Different answering methods were evaluated for the SI in the pilot study. Respondents preferred having the interviewer read the questions aloud and offer cards where participants would mark their answers, which the interviewer registered on a tablet. Participants felt comfortable answering the questions in public spaces with this method.

Data collection occurred in three phases, each with 2000 participants (6000 interviews in total). In order for the sample to be representative of the Brazilian adult population, the following procedures were adopted: (i) the total number of participants to be interviewed in each geographic region was calculated considering the proportion of the Brazilian population living in that area; (ii) the same calculation process was then used for each state and cities; (iii) cities, neighborhoods and interview venues (squares, crossroads, avenues, business streets, etc.) were randomly picked. A hundred and twenty-nine cities were drawn from a total of 5561 cities, with a probability proportional to their populations. This method allows each city and the groups of cities to have demographic representation in the sample.

The interviewees were randomly picked in public venues, where the data collection happened. Before SI questions were posed, participants were informed that the following questions regarded a current and important subject to society. Due to considerable stigma associated to gender identity discussions by political propaganda during 2018 Brazilian presidential elections, no further details were provided before SI’s questions. This prevented resistance against taking part in the study. No individuals turned down participating.

Interviewers were instructed to collect data from a previously agreed number of people of both genders as perceived by the interviewer (male or female) and of all age groups. In order to ensure representativeness, after having collected data from a number of individuals, socioeconomic status and geographic region were also used as selection criteria. Interviewers were trained regarding gender diversity and instructed to adopt a welcoming and impartial attitude towards the interviewees’ questions and answers.

### Ethical considerations

The study was approved by the Ethics Committee of São Paulo State University (UNESP), Medical School, Botucatu Campus, Brazil (protocol number: 2903853). All methods were carried out following relevant guidelines and regulations. Informed consent was obtained from all subjects before the interviews. Participants did not receive a financial incentive for taking part in the study.

### Measures

#### General instrument—GI

Participant’s sociodemographic characteristics were assessed with items regarding: geographic region where data was collected (1 = Southeast, 2 = South, 3 = Northeast, 4 = Midwest, 5 = North); urbanity (1 = capital city, 2 = metropolitan area, or 3 = countryside); age (in years); relationship status divided into 2 groups (1 = married/in a relationship, 2 = single, widowed or divorced/not in a relationship); level of education (1 = up to middle school, 2 = higher education); economically active population (EAP) (1 = EAP, 0 = not EAP); number of children; and social class (according to average family income) divided in 3 groups (1 = A/B, above US$ 1380.00/month; 2 = C between US$ 434.00 and US$ 760.00/month; 3 = D/E around US$ 182.00/month)^21^. US Dollar values considered the mean exchange rate in December 2018.

#### Specific instrument—SI

Participants’ gender identity was assessed through three questions with simple and objective wording to prevent misunderstandings. Question (Q)1: Which of the following options best describes how you currently feel? (1 = I feel I am a man; 2 = I feel I am a woman; 3 = I feel I am neither a man nor a woman). Q2: And what is the sex on your birth certificate? (1 = male; 2 = female; 3 = undetermined). Q3: Which of these situations do you most closely relate to? (1 = I was born male, but I have felt female since childhood; 2 = I was born female, but I have felt male since childhood; 3 = I was born male and I feel comfortable with my body; 4 = I was born female, and I feel comfortable with my body). Gender group classification of participants was defined by analyzing the answers to these 3 questions. Individuals that identified themselves with the gender assigned at birth and were gender-wise comfortable with their bodies were categorized as cisgender. Persons who identified with the binary gender opposite to their gender assigned at birth were categorized as transgender. When participants identified with neither binary genders and were gender-wise comfortable with their bodies were categorized as non-binary. For a detailed description of classification criteria, see Supplementary Table 1.

Apart from the three first SI questions, transgender individuals were asked two extra questions: Q3a (for Q3 = 1): Currently, you…: (1 = have a woman’s body; 2 = wish to have a woman’s body; 3 = do not wish to have a woman’s body). Q3b (for Q3 = 2): Currently, you…: (1 = have a man’s body; 2 = wish to have a man’s body; 3 = do not wish to have a man’s body). After this, respondents of Q3a or Q3b were asked the following questions to assess body-related distress: Q4a: Have you ever suffered for feeling your body is not congruent with how you feel? (1 = No distress; 2 = Some distress; 3 = Much distress). Q4b: And currently, do you suffer for feeling your body is not congruent with how you feel? (1 = No distress; 2 = Some distress; 3 = Much distress). For all questions in both GI and SI, the last answer options were: A = Do not know/do not understand the question; B = Refuse to answer.

### Statistical analysis

In order for analyses to be representative for the Brazilian adult population, data were weighted by geographic region, gender as perceived by the interviewer, age, social class and level of education. With the weighted sample, we assessed the association between gender and demographic characteristics of individuals using Pearson’s linear independence test and normal response model, followed by simple contrast comparisons. Distress due to body characteristics was assessed using Pearson’s linear independence test and the general linear model. Three logistic regression models were fit. The first one estimated the predictive relationship of gender identity on social class, adjusted for demographic covariates age (years), urbanity, country region, and education. The second one estimated the predictive relationship of gender identity on education, adjusted for age (years), urbanity, country region, and social class. The last one evaluated the predictive relationship of gender identity on relationship status, adjusted for social class, urbanity and education. All regression models were evaluated for the absence of multicollinearity, and goodness-of-fit. In all analyses, statistical significance was considered when p < 0.05. All analyses were performed using Statistical Package for the Social Sciences (SPSS) for Windows, version 22.0 (SPSS, Chicago, IL, USA).

## Results

Out of the 6000 interviews, for 70 interviewees (1.8% of the total sample), defining gender identity was not possible due to missing answers for the gender-related questions. The sample used in the analyses was, therefore, 5930 people (98.8%). In the sample, 40 (0.69%, 95% CI = 0.48 to 0.90) people were categorized as transgender, 20 (0.33%) were registered as male, and 20 (0.33%) as female; 71 (1.19%, CI 95% = 0.92 to 1.47) people were categorized as NBG, 33 (0.55%) were registered as male, and 38 (0.64%) as female. Mean age was significantly lower (p < 0.001) for TG people (32.9 ± 13.5) when compared to cisgender (42.2 ± 15.9) or NBG individuals (42.1 ± 16.5). The NBG group was less likely to be in a relationship compared to the cisgender group (33% vs. 48.7%). No further differences were found among the other sociodemographic variables (Table 1). It is important to note that, due to the small number of gender-diverse persons in our sample, the analyses associating gender identity and sociodemographic variables are preliminary.

**Table 1:** Weighted sociodemographic characteristics of each gender identity group (cisgender, transgender, and non-binary gender).

Variable	Gender group, n = 5930 (100%)									*P*
	Cisgender, n = 5819 (98.07%, CI 95% = 97.7 to 98.4)			Transgender, n = 40 (0.69%, CI 95% = 0.48 to 0.90)			Non-binary gender, n = 71 (1.19%, CI 95% = 0.92 to 1.47)			
	n (%)	CI 95%		n (%)	CI 95%		n (%)	CI 95%		
**Brazil’s region**										0.867
Southeast	2485 (43.7)	42.4	45.0	18 (47)	31.7	62.3	30 (45.6)	34.0	57.2	
South	908 (14.9)	14.0	15.8	6 (12.4)	2.3	22.5	12 (14.2)	6.1	22.3	
Northeast	1484 (25.7)	24.6	26.8	11 (27)	13.4	40.6	21 (29.3)	18.7	39.9	
Midwest	483 (7.7)	7.0	8.4	1 (2.5)	0.0	7.3	5 (6.5)	0.8	12.2	
North	460 (8)	7.3	8.7	4 (11.1)	1.5	20.7	3 (4.4)	0.0	9.2	
**Urbanity**										0.851
Capital	1501 (23.9)	22.8	25.0	13 (30.3)	16.2	44.4	19 (24.9)	14.8	35.0	
Metropolitan region	1106 (17.8)	16.8	18.8	8 (18.9)	6.9	30.9	14 (20)	10.7	29.3	
Countryside	3212 (58.3)	57.0	59.6	19 (50.8)	35.5	66.1	38 (55.1)	43.5	66.7	
**Education**										0.144
Up to high school	4969 (87.1)	86.2	88.0	38 (96.3)	90.5	102.1	62 (89.4)	82.2	96.6	
Higher education	850 (12.9)	12.0	13.8	2 (3.7)	0.0	9.5	9 (10.6)	3.4	17.8	
**Relationship status**										0.010^a^
In a relationship	2832 (48.7)	47.4	50.0	14 (35.3)	20.7	49.9	23 (33)	22.1	43.9	
Not in a relationship	2984 (51.3)	50.0	52.6	27 (64.7)	50.1	79.3	48 (67)	56.1	77.9	
**Belonging to EAP**	4161 (71.2)	70.0	72.4	29 (70.6)	56.7	84.5	54 (75.5)	65.5	85.5	0.752
**Social class**										0.177
A/B	1688 (24.1)	23.0	25.2	9 (17.3)	5.7	28.9	19 (21)	11.5	30.5	
C	2665 (48.2)	46.9	49.5	15 (38.1)	23.2	53.0	32 (48.8)	37.2	60.4	
D/E	1466 (27.7)	26.5	28.9	16 (44.6)	29.4	59.8	20 (30.2)	19.5	40.9	
**Mean age ± SD**	42.2 ± 15.9	42.0	42.8	32.9 ± 13.5	28.5	37.1	42.1 ± 16.5	38.3	46.5	< 0.001^b^
**Number of children ± SD**	1.6 ± 1.6	1.56	1.64	1.1 ± 1.8	0.48	1.52	1.6 ± 2.0	1.23	2.17	0.151

CI = confidence interval. All percentages and CIs were design-adjusted. EAP = economically active population. SD = standard deviation.

^a^Cisgender group ≠ non-binary gender group.

^b^Cisgender and non-binary gender groups ≠ transgender group.

### Relationship between gender group and sociodemographic characteristics

Regression analyses showed that gender identity did not predict the outcomes of social class and education (Supplementary Tables 2 and 3, respectively). However, relationship status could be predicted by gender identity (Table 2).

**Table 2:** Multinomial regression model evaluating the predictive effect of gender identity on relationship status.

OR of being in a relationship				
Variable	OR	CI95%		*p*
Intercept				0.012
**Gender group**				
Transgender	0.59	0.31	1.14	0.116
Non-binary	0.57	0.35	0.93	0.024^a^
Cisgender				
**Urbanity**				
Countryside	1.42	1.25	1.61	0.000
Metropolitan area	1.14	0.97	1.33	0.108
Capital city				
**Social class**				
D/E	0.44	0.37	0.51	0.000
C	0.61	0.53	0.70	0.000
A/B				
**Education**				
Up to high school	1.59	1.37	1.83	0.000
Higher education				

OR = odds ratio; CI = confidence interval.

^a^Non-binary gender group ≠ cisgender group.

### Perception of and distress due to gender-related body characteristics in transgender individuals

Not all individuals responded to every question in this section of the questionnaire (numbers and percentages of respondents for each question are shown in Table 3). Although not statistically significant, 7 (41.8%) TG women and 7 (39.6%) TG men reported having or wishing to have body characteristics of the gender with which they identify. Both TG women and men reported more past than current distress due to body characteristics.

**Table 3:** Perception of and distress due to gender-related body characteristics in transgender individuals.

	Transgender group				*p*
	Men		Women		
	n	%	n	%	
**Q3a/b Currently you…**					0.461
Have a body congruent with how you feel	5	28.8	2	15.9	
Wish to have a body congruent with how you feel	2	10.8	5	25.9	
Do not wish to change your body	12	60.4	11	58.2	
**Q4a Have you ever suffered for feeling your body is not congruent with how you feel?**					0.151
No distress	2	14.4	7	50.4	
Some distress	5	35.2	3	23.9	
Much distress	6	50.4	4	25.7	
**Q4b And nowadays, do you suffer for feeling your body is not congruent with how you feel?**					0.200
No distress	8	64.5	10	74.3	
Some distress	1	6.7	3	19.8	
Much distress	4	28.7	1	5.8	

All percentages were design-adjusted. Only individuals who answered the questions were included in the analysis (i.e., those who refused to answer or reported not understanding the question were not considered).

## Discussion

This is the first study to assess the proportion of gender diversity in a South American country. It was conducted in a representative sample of the adult population in Brazil. Gender diversity was observed in roughly 1.9% of the sample. Persons identifying as TG represented 0.69%, and 1.19% fell into the NBG identity group. Considering that the Brazilian adult population (18 years or older) at the time of data collection was approximately 158,000,000^20^, we estimate that 1,090,200 Brazilians may identify as TG, and 1,880,200 as NBG. Consequently, amounting to almost 3 million people identifying as gender-diverse in Brazil.

Most of the population studies done in the US, which estimated the proportion of adult people who self-identify as TG found percentages similar to our findings. Conron et al. assessed a representative household sample between 2007 and 2009 that participated in a telephone health survey in Massachusetts, US, and found 0.5% of the respondents self-identified as TG^22^. In 2011, Gates et al. estimated that the proportion of TG individuals in California was 0.1% of the population, based on data collected in the 2003 LGBT CA Tobacco Survey and in the 2009 Health Interview Survey^23^. Using data from an annual cross-sectional telephone survey held in 2014, Flores et al. estimated that 0.6% of US Americans self-identified as TG^12^. Similar findings were reported by Crissman et al. using the same database to investigate demographic aspects of TG and non-TG populations: 0.53% of US Americans were TG^14^. In a study using data from probabilistic samples collected from 12 population-based studies held in the US between 2007 and 2015, Meerwijk and Sevelius found that for every 390 people, one is TG, and this proportion has increased in the last decade^24^. In a longitudinal cohort population-based study, that included adults in Stockholm, Ahs and colleagues showed that 2.8% of the population wished to live or be treated as a person of the other sex, 2.3% felt like someone of another sex, and 0.5% of the population reported wishing to have gender-affirming surgery or hormone therapy^10^.

In our study, we observed the same proportion of TG people in both binary genders. Other population studies found a higher proportion of trans women amongst those who identified as TG^12,14^. This difference indicates that broad population studies may lack power to accurately evaluate gender ratios within the TG binary population.

In a US study using a large prospective cohort of young adults, Reisner et al. used a two-step method to infer the gender identity of interviewed participants. The first question assessed participants’ sex (in their birth certificate), and the second one assessed participants’ current gender identity. The authors found that 0.33% of respondents were TG^13^. In another study, Reisner et al. advised that health surveys including gender minorities use the two-step method to count TG and NBG individuals^25^. Bauer et al., on the other hand, suggested a multidimensional model with the first two questions similar to the two-step method, and a third question regarding which gender people identified as in daily life^26^. Neither of the above options would be entirely suitable for the Brazilian population, since we identified in a pilot study that respondents were not familiar enough with terms involving gender identity we, therefore, developed a questionnaire based on current gender identity definitions^1^ with easy-to-understand language. By using this method, we were able to categorize the gender identity of almost all respondents (98.8% of the sample). It is worth noting that all population studies that investigate the proportion of gender-diverse people were conducted in countries with higher human development indices and/or in countries that are more tolerant of gender diversity^9,27^. It is also reasonable to believe that specific terminology (e.g.: cisgender, TG, NBG) may be an obstacle when assessing populations that are less aware of the subject.

The proportion of GNC people in Belgium was investigated by Van Caenegem and colleagues, with population-based surveys carried out in person or over the internet. The authors found gender ambivalence (feeling like both men and women) in 2.2% of adult males, and 1.9% of adult females, whereas gender incongruence was observed in 0.7% of adult males, and 0.6% of adult females^3^. A similar study conducted by Kuyper and Wijsen in the Netherlands showed that 1.1% of adult males and 0.8% of adult females reported having an incongruent gender identity, and 4.6% of adult males and 3.2% of adult females reported gender ambiguity^5^. In previous studies, similar to the one conducted by Ahs and colleagues in Sweden^10^, broader questionnaires with multiple choice questions (rather than just yes or no) were used that showed sensitivity in identifying incongruence and gender variations from the respondents. In the above studies, the proportion of GNC individuals was higher than what we found for NBG in our study. However, using such methods in the present study would be impractical, as our interviews were held in public, possibly causing respondents considerable discomfort.

Thirteen participants who answered they felt their gender was different from the one assigned at birth in Q3 (32.5% of the TG group, 11 trans women and 2 trans men) also answered “I feel neither a man nor a woman” in Q1. This may indicate a considerable number of TG individuals with possible non-binary or gender-nonconforming identities. Reisner et al. mentioned that a great part of research on TG people’s health treated these individuals as a homogeneous population, or in the best cases, categorized them in a binary way—TG man/male, TG woman/female. The authors also advised that both binary and non-binary gender identity choices are included in questionnaires used in public health research^28^. Motmans, Nieder and Bouman highlighted the importance of considering the group of people who do not identify as binary TG^29^. Gender identities that go beyond binary are gaining more recognition among the general population^30^. However, only 11 studies were selected in a recent review on the health of non-binary people conducted by Scandurra and colleagues^31^. Future studies on the field should be inclusive of this population and favor questions about gender identity that are composed of multiple choices, instead of just the binary genders.

Our preliminary results found that TG people are on average younger than cisgender and NBG groups. Other population studies showed that younger people tend to identify more as gender-diverse than older people^22,24^. On the other hand, Brazil is the country with the worst rates of violence against TG people in the world^32^. The lower age average in the TG group may be explained by the group’s shorter life expectancy. We also found that NBG people are less likely to be in a relationship when compared to the cisgender group. Further studies are needed to shed light on the reasons for the lower likelihood of being in a relationship for NBG people. Other population studies show that TG people tend to be in lower social classes, and have lower levels of education^14,22^. In our study, we were unable to replicate such associations, but bigger samples may allow us to detect these differences.

Considering the similar distribution of cisgender versus transgender populations across Brazilian geographic regions, we stress the importance of public health policies that are inclusive of gender diversity throughout the country. To corroborate the urgency to do so, we found that among TG people, 15 (39.5%) have or wish to have body characteristics that belong to the gender with which they identify. Eighty-five percent of trans men and 50% of trans women reported having suffered from feeling that their body was not congruent with how they felt. On the other hand, around 60% of TG people reported not wishing to have body characteristics of the opposite binary gender, maybe reinforcing that not all TG people want physical intervention either by hormones or surgery^33^. We do not have any further data to explore the aspects of this finding, including circumstances of physical conformity linked to the comprehension of and/or prejudice against the condition of being TG.

In this study, we were unable to confirm that individuals would identify themselves in the same way that we had classified them within the three gender identity options (cisgender, TG and NBG) as categorized in the questionnaire. It is also worth noting that some options in question three (Q3) referred to feeling different from the assigned gender since childhood. This could exclude TG people who started feeling they were of a different gender at later stages in life. However, the respondents were not asked to choose the situation that described them fully, but rather the one that best described the way they feel. Race/ethnicity information was also not collected. The research institute responsible for data collection usually does not include this item^34^, due to the high variability in the perception of race in Brazil. Although Brazilian birth certificates only allow two options for sex, Q2 of our questionnaire offered the options “male”, “female”, and “undetermined” as a means of including people with sex development variations. Following our method of gender classification based on three questions, reporting undetermined sex would pose a challenge. However, only one individual chose that option, who was excluded from the analysis due to not responding another of the three questions used for gender classification^26^.

Since gender identity may be fluid over time^28^, our results only describe the current gender identity of the participants. Other aspects of gender diversity were not captured in the questionnaire and therefore, results may underestimate the proportion of people who have such experiences. It is not our aim to oversimplify gender-diverse people into two groups (TG and NBG), but rather present the first picture of Brazilians who identify as TG or as NBG, bearing in mind that future studies will paint more hues regarding the population’s gender variations.

## Supplementary Information

Supplementary Information.
