# Supplementary material for: Proportion of people identified as transgender and non-binary gender in Brazil
Source: Sci Rep. 2021 Jan 26;11:2240. doi: 10.1038/s41598-021-81411-4 (PMC7838397; doi:10.1038/s41598-021-81411-4)
Supplement: Supplementary file 1 — Supplementary Information. [file 41598_2021_81411_MOESM1_ESM.docx]

**Proportion of people identified as transgender and non-binary gender in Brazil**

**Supplementary information**

Giancarlo Spizzirri^1,*^, Raí Eufrásio^2^, Maria Cristina Pereira Lima^3^, Hélio Rubens de Carvalho Nunes^4^, Baudewijntje P.C. Kreukels^5^, Thomas D. Steensma^5^, Carmita Helena Najjar Abdo^1^

^1^ Department of Psychiatry, Faculdade de Medicina FMUSP, Universidade de Sao Paulo, SP, BR.

^2^Independent researcher, São Paulo, SP, Brazil.

^3^Department of Neurology, Psychology and Psychiatry, Botucatu Medical School, Universidade Estadual Paulista (UNESP), Botucatu (SP), Brazil.

^4^Programa de Pós-Graduação em Enfermagem, Universidade Estadual Paulista (UNESP), Botucatu (SP), Brazil.

^5^Department of Medical Psychology, Center of Expertise on Gender Dysphoria, Amsterdam University Medical Centers, location VU, Amsterdam, The Netherlands NL.

* *Correspondence to:* [giancki@uol.com.br](mailto:giancki@uol.com.br) (GS)

**Supplementary Tables**

**Supplementary Table 1. Gender categorization (cisgender man/woman, transgender man/woman or non-binary gender) following the association between answers to Specific Instrument’s Q1, Q2, and Q3.**

| Gender Group | Question 1 (Q1). Which of the following best describes how you currently feel? | Question 2 (Q2). And what sex is registered on your birth certificate? | Question 3 (Q3). Which of these situations best describes you? | n |
| --- | --- | --- | --- | --- |
| Cisgender man | 1 = I feel I am a man | 1 = Male | 3 = I was born man and I am comfortable with my body | 2708 |
|  | A = Do not know, or B = Refuses to answer | 1 = Male | 3 = I was born man and I am comfortable with my body | 8 |
|  | 1 = I feel I am a man | 1 = Male | A = Do not know, or B = Refuses to answer | 42 |
| Total | | | | 2758 |
| Cisgender woman | 2 = I feel I am a woman | 2 = Female | 4 = I was born woman, and I feel comfortable with my body | 3019 |
|  | A = Do not know, or B = Refuses to answer | 2 = Female | 4 = I was born woman, and I feel comfortable with my body | 12 |
|  | 2 = I feel I am a woman | 2 = Female | A = Do not know, or B = Refuses to answer | 30 |
| Total | | | | 3061 |
| Cisgender total | | | | 5818 |
| Transgender man | 1 = I feel I am a man | 2 = Female | 2 = I was born female, but I have felt male since childhood | 16 |
|  | 1 = I feel I am a man | 2 = Female | 3 = I was born man and I am comfortable with my body | 2 |
|  | 3 = I feel neither a man nor a woman | 2 = Female | 2 = I was born female, but I have felt male since childhood | 2 |
|  | A = Do not know, or B = Refuses to answer | 2 = Female | 2 = I was born female, but I have felt male since childhood | 0 |
| Total | | | | 20 |
| Transgender woman | 2 = I feel I am a woman | 1 = Male | 1 = I was born male, but I have felt female since childhood | 8 |
|  | 2 = I feel I am a woman | 1 = Male | 4 = I was born woman, and I feel comfortable with my body | 0 |
|  | 3 = I feel neither a man nor a woman | 1 = Male | 1 = I was born male, but I have felt female since childhood | 11 |
|  | A = Do not know, or B = Refuses to answer | 1 = Male | 1 = I was born male, but I have felt female since childhood | 1 |
| Total | | | | 20 |
| Transgender total | | | | 40 |
| Non-binary | 3= I feel neither a man nor a woman | 1 = Male | 3 = I was born man and I feel comfortable with my body | 32 |
|  | 3= I feel neither a man nor a woman | 2 = Female | 4 = I was born woman and I feel comfortable with my body | 36 |
|  | 3= I feel neither a man nor a woman | 1 = Male | A = Do not know, or B = Refuses to answer | 1 |
|  | 3= I feel neither a man nor a woman | 2 = Female | A = Do not know, or B = Refuses to answer | 2 |
| Non-Binary total | | | | 71 |
| Grand Total | | | | 5930 |

Notes: the number of individuals in each specific group, the total for each gender group, as well as the grand total are shown in the last column.

**Supplementary Table 2. Multinomial regression model evaluating the predictive effect of gender identity on social class.**

| OR of belonging to social class C, having A/B as reference | | | | |
| --- | --- | --- | --- | --- |
| Variable | OR | CI95% | | *p* |
| Intercept |  |  |  | .000 |
| Age (years) | 1.00 | 0.99 | 1.00 | .399 |
| Gender Group |  |  |  |  |
| Transgender | 0.91 | 0.38 | 2.22 | .841 |
| Non-binary | 1.04 | 0.56 | 1.93 | .898 |
| Cisgender |  |  |  |  |
| Urbanity |  |  |  |  |
| Countryside | 1.28 | 1.09 | 1.49 | .002 |
| Metropolitan area | 1.17 | 0.97 | 1.43 | .110 |
| Capital city |  |  |  |  |
| Country region |  |  |  |  |
| North | 2.08 | 1.55 | 2.80 | .000 |
| South | 1.08 | 0.90 | 1.29 | .415 |
| Northeast | 1.70 | 1.42 | 2.05 | .000 |
| Midwest | 1.07 | 0.84 | 1.35 | .594 |
| Southeast |  |  |  |  |
| Education |  |  |  |  |
| Up to high school | 5.93 | 5.13 | 6.86 | .000 |
| Higher education |  |  |  |  |
|  |  |  |  |  |
| OR of belonging to social class D/E, having A/B as reference | | | | |
| Variable | OR | CI95% | | *p* |
| Intercept |  |  |  | .000 |
| Age (years) | 1.01 | 1.00 | 1.01 | .002 |
| Gender Group |  |  |  |  |
| Transgender | 2.03 | 0.78 | 5.30 | .150 |
| Non-binary | 1.18 | 0.57 | 2.48 | .654 |
| Cisgender |  |  |  |  |
| Urbanity |  |  |  |  |
| Countryside | 2.97 | 2.41 | 3.66 | .000 |
| Metropolitan area | 2.33 | 1.80 | 3.02 | .000 |
| Capital city |  |  |  |  |
| Country region |  |  |  |  |
| North | 6.45 | 4.62 | 8.94 | .000 |
| South | 0.81 | 0.62 | 1.03 | .084 |
| Northeast | 5.93 | 4.80 | 7.32 | .000 |
| Midwest | 1.35 | 0.98 | 1.85 | .064 |
| Southeast |  |  |  |  |
| Education |  |  |  |  |
| Up to high school | 37.22 | 26.27 | 52.73 | .000 |
| Higher education |  |  |  |  |

Notes: OR = odds ratio; CI = confidence interval.

**Supplementary table 3. Multinomial regression model evaluating the predictive effect of gender identity on education.**

| OR of having studied up to high school, having higher education as reference | | | | |
| --- | --- | --- | --- | --- |
| Variable | OR | CI95% | | *p* |
| Intercept |  |  |  | .000 |
| Age (years) | 1.02 | 1.01 | 1.02 | .000 |
| Gender Group |  |  |  |  |
| Transgender | 2.20 | 0.77 | 6.32 | .143 |
| Non-binary | 1.13 | 0.58 | 2.18 | .726 |
| Cisgender |  |  |  |  |
| Urbanity |  |  |  |  |
| Countryside | 1.78 | 1.51 | 2.09 | .000 |
| Metropolitan area | 1.67 | 1.36 | 2.10 | .000 |
| Capital city |  |  |  |  |
| Country region |  |  |  |  |
| North | 0.91 | 0.68 | 1.21 | .508 |
| South | 1.22 | 1.00 | 1.50 | .050 |
| Northeast | 1.08 | 0.89 | 1.31 | .434 |
| Midwest | 0.92 | 0.72 | 1.19 | .534 |
| Southeast |  |  |  |  |
| Social class |  |  |  |  |
| D/E | 36.61 | 25.86 | 51.83 | .000 |
| C | 5.96 | 5.15 | 6.90 | .000 |
| A/B |  |  |  |  |

Notes: OR = odds ratio; CI = confidence interval.
